# Supplementary material for: Ocean acidification increases susceptibility to sub-zero air temperatures in ecosystem engineers and limits poleward range shifts
Source: eLife. 2023 Apr 11;12:e81080. doi: 10.7554/eLife.81080 (PMC10129327; doi:10.7554/eLife.81080)
Supplement: Supplementary file 1. [file elife-81080-supp1.docx]

| GLM Model  (Binomial distribution) | Estimate | s.e | z-value | *p*-value |
| --- | --- | --- | --- | --- |
| Subtidal *Mytilus galloprovincialis* (Explained deviance = 95.37%) | | | | |
| Intercept | 11.96 | 1.81 | 6.59 | < 0.0001 |
| Air temperature | 1.58 | 0.24 | 6.63 | < 0.0001 |
| Low pH | -1.42 | 0.50 | -2.82 | < 0.0001 |
|  |  |  |  |  |
| Subtidal *Mytilus trossulus* (Explained deviance = 91.49%) | | | | |
| Intercept | 8.11 | 1.13 | 7.20 | < 0.0001 |
| Air temperature | 0.88 | 0.13 | 6.84 | < 0.0001 |
| Low pH | -1.31 | 0.43 | -3.05 | < 0.0001 |
|  |  |  |  |  |
| Intertidal *Mytilus trossulus* (Explained deviance = 93.94%) | | | | |
| Intercept | 12.71 | 1.83 | 6.93 | < 0.0001 |
| Air temperature | 1.21 | 0.17 | 6.79 | < 0.0001 |
| Low pH | -3.29 | 0.70 | -4.73 | < 0.0001 |
|  |  |  |  |  |
| Survival among mussel categories (Explained deviance = 90.7%) | | | | |
| Intercept | 7.08 | 0.79 | 11.15 | < 0.0001 |
| Air temperature | 0.99 | 0.10 | 11.32 | < 0.0001 |
| Intertidal *Mytilus trossulus* | 1.80 | 0.34 | 5.97 | < 0.0001 |
| Subtidal *Mytilus trossulus* | 1.25 | 0.32 | 4.45 | < 0.0001 |
|  |  |  |  |  |
