# Supplementary material for: Ocean acidification increases susceptibility to sub-zero air temperatures in ecosystem engineers and limits poleward range shifts
Source: eLife. 2023 Apr 11;12:e81080. doi: 10.7554/eLife.81080 (PMC10129327; doi:10.7554/eLife.81080)
Supplement: Supplementary file 2. [file elife-81080-supp2.docx]

|  | *M. trossulus* - intertidal | | *M. trossulus* - subtidal | | *M.* *galloprovincialis* - subtidal | |
| --- | --- | --- | --- | --- | --- | --- |
| Metabolites | Acidified | Control | Acidified | Control | Acidified | Control |
| Acetate | 0.41±0.09 | 0.42±0.16 | 0.46±0.09 | 0.36±0.12 | 0.40±0.16 | 0.50±0.16 |
| Acetoacetate | 0.2±0.13 | 0.12±0.12 | 0.05±0.07 | 0.14±0.15 | 0.11±0.15 | 0.20±0.22 |
| Alanine | 2.43±0.78 | 1.82±0.90 | 1.79±1.68 | 1.61±0.56 | 3.12±1.98 | 3.08±1.53 |
| AMP | 0.47±0.47 | 0.37±0.12 | 0.54±0.22 | 0.55±0.45 | 0.70±0.36 | 0.56±0.24 |
| Arginine | 0.46±1.04 | 0 | 0 | 0 | 0.36±0.8 | 0 |
| Asparagine | 0 | 0 | 0.21±0.48 | 0 | 0 | 0 |
| Aspartate | 2.35±0.62 | 2.36±0.45 | 2.72±1.26 | 2.90±0.45 | 2.24±1.4 | 2.75±1.60 |
| Betaine | 10.09±1.58 | 12.0±1.82 | 12.93±3.04 | 11.70±1.81 | 13.15±0.61 | 15.03±2.26 |
| Glutamate | 2.52±4.60 | 0.35±0.53 | 0.56±0.87 | 0 | 0.13±0.28 | 0 |
| Glycine | 6.29±3.90 | 6.70±2.45 | 4.79±4.30 | 6.69±4.24 | 6.41±4.76 | 8.75±4.52 |
| Guanidoacetate | 0.18±0.29 | 0.025±0.06 | 0.16±0.26 | 0 | 0.07±0.09 | 0 |
| Lactate | 0.22±0.16 | 0.38±0.34 | 0.04±0.06 | 0.16±0.10 | 0.47±0.35 | 0.53±0.24 |
| Lysine | 0.15±0.34 | 0 | 0 | 0 | 0.25±0.56 | 0.41±0.91 |
| Malate | 0.42±0.93 | 0 | 1.04±1.44 | 0 | 0.36±0.81 | 0 |
| Malonate | 2.24±0.67 | 1.70±1.25 | 1.40±0.91 | 1.54±0.88 | 2.05±1.84 | 1.82±1.28 |
| Proline | 0.55±0.76 | 0 | 0 | 0.27±0.60 | 0.67±0.92 | 0 |
| Succinate | 0.31±0.10 | 0.16±0.11 | 0.36±0.26 | 0.35±0.24 | 0.30±0.24 | 0.41±0.21 |
| Taurine | 13.52±1.66 | 15.36±1.54 | 17.07±4.11 | 15.85±1.42 | 17.34±2.14 | 19.91±2.62 |
| Trimethylamine | 1.21±0.85 | 1.62±1.10 | 1.33±1.59 | 2.04±0.90 | 2.49±1.05 | 2.43±1.19 |
| TMAO | 1.24±0.76 | 1.46±0.30 | 1.93±1.41 | 1.87±1.01 | 1.32±0.50 | 2.93±1.46 |
| β-Alanine | 0.87±0.34 | 0.68±0.56 | 0.26±0.43 | 0.54±0.43 | 0.55±0.67 | 0.45±0.35 |
