# Supplementary material for: Ocean acidification increases susceptibility to sub-zero air temperatures in ecosystem engineers and limits poleward range shifts
Source: eLife. 2023 Apr 11;12:e81080. doi: 10.7554/eLife.81080 (PMC10129327; doi:10.7554/eLife.81080)
Supplement: Supplementary file 3. [file elife-81080-supp3.docx]

|  | Control | | |  | Acidified | | |
| --- | --- | --- | --- | --- | --- | --- | --- |
| Incubator | 1 | 2 | 3 |  | 4 | 5 | 6 |
| pH | 7.84 ± 0.19 | 7.92 ± 0.12 | 7.87 ± 0.13 |  | 7.55 ± 0.03 | 7.49 ± 0.05 | 7.51 ± 0.06 |
| Temperature | 6.67 ± 0.27 | 6.95 ± 0.36 | 6.86 ± 0.19 |  | 7.17 ± 0.26 | 7.08 ± 0.54 | 6.89 ± 0.13 |
