# Supplementary material for: Ocean acidification increases susceptibility to sub-zero air temperatures in ecosystem engineers and limits poleward range shifts
Source: eLife. 2023 Apr 11;12:e81080. doi: 10.7554/eLife.81080 (PMC10129327; doi:10.7554/eLife.81080)
Supplement: Supplementary file 4. [file elife-81080-supp4.docx]

|  | |  | | PSU | pH | | | Alkalinity | pCO_2_ | | | DIC | Ω aragonite | | | Ω calcite |
| --- | --- | --- | --- | --- | --- | --- | --- | --- | --- | --- | --- | --- | --- | --- | --- | --- |
| Control | Start | | 22.43 ± 0.06 | | | 8.09 *±* 0.16 | 1338.04 *±* 23.18 | | | 242.01 103.32 | 1254.45 *±* 21.50 | | | 0.90 *±* 0.28 | 1.49 *±* 0.46 | |
|  | End | | 23.23 *±* 0.32 | | | 8.13 *±* 0.05 | 882.35 *±* 104.65 | | | 134.82 *±* 30.91 | 807.24 *±* 105.68 | | | 0.62 *±* 0.07 | 1.03 *±* 0.12 | |
| Acidified | Start | | 22.53 *±* 0.06 | | | 7.58 *±* 0.21 | 1498.84 *±* 94.29 | | | 968.71 *±* 505.71 | 1412.77 *±* 88.94 | | | 0.36 *±* 0.15 | 0.59 *±* 0.24 | |
|  | End | | 23.57 *±* 0.23 | | | 7.53 *±* 0.18 | 1549.26 *±* 166.31 | | | 1088.12 *±* 469.34 | 1571.05 *±* 171.16 | | | 0.34 *±* 0.16 | 0.55 *±* 0.26 | |

**Abbreviations:** PSU, Practical salinity unit; pCO_2_, Partial pressure of carbon dioxide; DIC, Dissolved inorganic carbon**. Symbols:** Ω, Omega.
