# Supplementary material for: Ocean acidification increases susceptibility to sub-zero air temperatures in ecosystem engineers and limits poleward range shifts
Source: eLife. 2023 Apr 11;12:e81080. doi: 10.7554/eLife.81080 (PMC10129327; doi:10.7554/eLife.81080)
Supplement: Supplementary file 5. — Bold numbers indicate anomalous values. [file elife-81080-supp5.docx]

| Treatment | Incubator | DIC (µmol/kg) | TA (µmol/kg) | pCO2 (µatm) | pH (total) | Aragonite  (Ω) | Calcite  (Ω) |
| --- | --- | --- | --- | --- | --- | --- | --- |
| Control (start) | 3 | 1230.64 | 1335.34 | 176.67 | 8.19 | 1.07 | 1.76 |
| Control (start) | 1 | 1260.26 | 1362.46 | 188.24 | 8.17 | 1.06 | 1.74 |
| Control (start) | 2 | 1272.46 | 1316.33 | 361.12 | 7.90 | 0.58 | 0.96 |
| **Control (end)** | 3 | **916.80** | **986.24** | **170.48** | 8.08 | 0.63 | 1.04 |
| **Control (end)** | 2 | **705.92** | **776.96** | **115.85** | 8.13 | 0.55 | 0.90 |
| **Control (end)** | 1 | **798.98** | **883.86** | **118.12** | 8.17 | 0.69 | 1.13 |
| Acidified (start) | 5 | 1421.88 | 1433.95 | 621.42 | 7.71 | 0.42 | 0.70 |
| Acidified (start) | 6 | 1516.80 | 1455.56 | 1548.91 | 7.34 | 0.19 | 0.31 |
| Acidified (start) | 4 | 1599.63 | 1607.00 | 735.82 | 7.69 | 0.45 | 0.75 |
| Acidified (end) | 5 | 1620.13 | 1637.31 | 678.28 | 7.73 | 0.52 | 0.85 |
| Acidified (end) | 4 | 1712.32 | 1653.04 | 1600.13 | 7.37 | 0.24 | 0.39 |
| Acidified (end) | 6 | 1380.71 | 1357.44 | 985.94 | 7.49 | 0.26 | 0.42 |

**Abbreviations:** DIC, Dissolved inorganic carbon; TA, Total alkalinity; pCO_2_, Partial pressure of carbon dioxide. **Symbols:** Ω, Omega.
