# Supplementary material for: Ocean acidification increases susceptibility to sub-zero air temperatures in ecosystem engineers and limits poleward range shifts
Source: eLife. 2023 Apr 11;12:e81080. doi: 10.7554/eLife.81080 (PMC10129327; doi:10.7554/eLife.81080)
Supplement: Supplementary file 6. — The molar percentage of saturated fatty acids (SFA), monounsaturated fatty acids (MUFA), and polyunsaturated fatty acids (PUFA) is presented. [file elife-81080-supp6.docx]

|  | Intertidal - *M. trossulus* | | Subtidal *- M. trossulus* | | Subtidal - *M.* *galloprovincialis* | |
| --- | --- | --- | --- | --- | --- | --- |
| Fatty acid | Acidified | Control | Acidified | Control | Acidified | Control |
| 14:0 | 0.40±0.07 | 0.4±0.09 | 0.54±0.24 | 0.3±0.05 | 0.62±0.14 | 0.37±0.07 |
| 15:0 | 0.52±0.04 | 0.51±0.05 | 0.69±0.21 | 0.41±0.05 | 0.63±0.26 | 0.44±0.09 |
| 15:0-iso | 0.07±0.06 | 0.09±0.1 | 0.2±0.19 | 0.16±0.06 | 0.00 | 0.08±0.05 |
| 16:0 | 9.53±0.89 | 8.72±1.21 | 16.56±6.62 | 10.21±1.21 | 12.95±5.17 | 8.83 ±1.44 |
| 17:0 | 0.45±0.05 | 0.43±0.04 | 0.88±0.58 | 0.38±0.05 | 0.61±0.28 | 0.41±0.09 |
| 17:0-iso | 0.3±0.07 | 0.29±0.05 | 0.29±0.13 | 0.12±0.02 | 0.24±0.07 | 0.19±0.02 |
| 17:0-ante | 0.17±0.03 | 0.14±0.02 | 0.19±0.09 | 0.08±0.01 | 0.17±0.06 | 0.12±0.03 |
| 18:0 | 2.57±0.34 | 2.53±0.17 | 4.30±1.98 | 2.28±0.38 | 3.38±1.26 | 2.53±0.67 |
| 18:0-iso | 0.11±0.01 | 0.1±0.02 | 0.21±.011 | 0.08±0.01 | 0.21±0.07 | 0.17±0.03 |
| **∑SFA** | **14.12±1.31** | **13.2±1.27** | **23.86±9.57** | **14.03±1.53** | **18.81±7.26** | **13.12±2.3** |
| 16:1ω7 | 1.01±0.1 | 1.2±0.22 | 1.58±0.33 | 0.97±0.19 | 1.71±0.33 | 1.07±0.27 |
| 17:1ω7 | 1.71±0.63 | 4.07±4.16 | 1.94±1.14 | 1.43±1.1 | 2.26±1.63 | 2.04±2.15 |
| 18:1ω7 | 1.48±0.12 | 1.37±0.37 | 1.91±0.52 | 1.21±0.2 | 2.08±0.58 | 1.5±0.34 |
| 18:1ω9 | 1.04±0.16 | 1.01±0.24 | 1.23±0.32 | 1.07±0.29 | 1.2±0.33 | 0.97±0.26 |
| 20:1ω7 | 0.65±0.1 | 0.60±0.14 | 0.75±0.2 | 0.34±0.06 | 0.97±0.27 | 0.61±0.12 |
| 20:1ω9 | 4.31±0.3 | 4.12±0.46 | 6.21±1.13 | 4.86±0.61 | 5.43±1.32 | 4.37±0.48 |
| 20:1ω11 | 0.67±0.14 | 0.74±0.19 | 0.74±0.15 | 0.53±0.08 | 1.14±0.19 | 0.82±0.15 |
| **∑MUFA** | **10.87±1.1** | **13.13±3.68** | **14.36±3.33** | **10.41±1.02** | **14.78±1.65** | **11.39±1.63** |
| 18:2ω6 | 0.91±0.1 | 0.94±0.35 | 1.46±0.67 | 1.31±0.25 | 0.79±0.36 | 0.64±0.14 |
| 18:3ω3 | 1.12±0.07 | 1.21±0.5 | 1.04±0.57 | 1.52±0.19 | 0.31±0.1 | 0.36±0.09 |
| 18:4ω3 | 0.76±0.19 | 1.07±0.41 | 0.67±0.25 | 1.09±0.28 | 0.39±0.13 | 0.33±0.11 |
| 20:2ω6 | 9.41±0.76 | 8.82±1.05 | 11.96±1.21 | 9.87±0.58 | 9.78±1.79 | 8.67±1.41 |
| 20:3ω6 | 0.11±0.02 | 0.12±0.04 | 0.00 | 0.09±0.02 | 0.16±0.03 | 0.12±0.02 |
| 20:4ω6 | 9.7±1.1 | 9.81±0.9 | 3.76±2.61 | 6.07√0.76 | 6.95±2.43 | 9.7±1.37 |
| 20:5ω3 | 9.78±1.15 | 10.74±1.97 | 4.87±3.59 | 12.19±1.05 | 7.0±2.98 | 10.21±1.68 |
| 22:2-NMI | 11.54±0.84 | 10.86±1.5 | 12.72±0.95 | 9.55±0.66 | 13.53±1.5 | 12.19±.1.14 |
| 22:6 ω3 | 14.13±0.99 | 13.73±0.69 | 6.36±4.86 | 16.22±0.48 | 9.7±4.61 | 15.88±2.06 |
| PUFA | 1.19±0.23 | 0.98±0.1 | 1.51±0.7 | 2.36±0.42 | 0.72√0.07 | 0.81±0.17 |
| PUFA1 | 2.62±0.52 | 2.47±0.25 | 2.84±1.49 | 4.06±0.34 | 1.37±0.42 | 1.72±0.42 |
| PUFA2 | 0.93±0.26 | 0.86±0.23 | 0.4±0.11 | 0.38±0.08 | 0.91±0.33 | 1.09±0.12 |
| PUFA3 | 0.68±0.11 | 0.64±0.07 | 0.46±0.1 | 0.52±0.05 | 0.77±0.18 | 1.14±0.22 |
| PUFA4 | 11.54±0.8 | 10.86±1.5 | 12.72±0.95 | 9.55±0.66 | 13.52±1.5 | 12.19±1.14 |
| **∑PUFA** | **75.0±2.05** | **73.67±2.93** | **61.78±12.27** | **75.56±1.65** | **66.4±8.57** | **75.49±2.1** |

**Abbreviations:** 14:0, Myristic acid; 15:0, Pentadecylic acid; 16:0, Palmitic acid; 17:0, Margaric acid; 18:0, Stearic acid; 16:1ω7, Palmitoleic acid; 17:1ω7, 10Z-Heptadecenoic acid; 18:1ω7, Vaccenic acid; 18:1ω9, Elaidic acid; 20:1ω7, Paullinic acid; 20:1ω9, Gondoic acid; 20:1ω11, Gadoleic acid; 18:2ω6, Linolelaidic acid; 18:3ω3, γ-Linolenic acid; 18:4ω3, Stearidonic acid; 20:2ω6, Docosadienoic acid; 20:3ω6, Dihomo-γ-linolenic acid; 20:4ω6, Arachidonic acid; 20:5ω3, Eicosapentaenoic acid; 22:2-NMI, Behenate non-methylene-interrupted; 22:6ω3, Docosahexaenoic acid.
